# Decoding Multiple Sound-Categories in the Auditory Cortex by Neural Networks: An fNIRS Study

**DOI:** 10.3389/fnhum.2021.636191

**Published:** 2021-04-28

**Authors:** So-Hyeon Yoo, Hendrik Santosa, Chang-Seok Kim, Keum-Shik Hong

**Affiliations:** ^1^School of Mechanical Engineering, Pusan National University, Busan, South Korea; ^2^Department of Radiology, University of Pittsburgh, Pittsburgh, PA, United States; ^3^Department of Cogno-Mechatronics Engineering, Pusan National University, Busan, South Korea

**Keywords:** functional near-infrared spectroscopy (fNIRS), long short-term memories (LSTMs), auditory cortex, decoding, deep learning

## Abstract

This study aims to decode the hemodynamic responses (HRs) evoked by multiple sound-categories using functional near-infrared spectroscopy (fNIRS). The six different sounds were given as stimuli (English, non-English, annoying, nature, music, and gunshot). The oxy-hemoglobin (HbO) concentration changes are measured in both hemispheres of the auditory cortex while 18 healthy subjects listen to 10-s blocks of six sound-categories. Long short-term memory (LSTM) networks were used as a classifier. The classification accuracy was 20.38 ± 4.63% with six class classification. Though LSTM networks’ performance was a little higher than chance levels, it is noteworthy that we could classify the data subject-wise without feature selections.

## Introduction

Recognizing sound is one of the important senses in everyday life. People are always exposed to a variety of sounds, and they can know what sound it is without being conscious. This ability allows people to avoid various dangers and facilitates communication with others. The auditory stimulus that enters through the outer ear is transmitted to the auditory cortex through the auditory nerve. It is clear that the temporal cortex is activated differently by different sounds. Neural responses in the auditory cortices have been studied using diverse modalities like electroencephalography (EEG; Wong et al., 2007; Hill and Scholkopf, 2012; Liu et al., 2015), magnetoencephalography (MEG; Hyvarinen et al., 2015), eletrocorticogram (ECoG; Pasley et al., 2012; Herff et al., 2015), functional magnetic resonance imaging (fMRI; Wong et al., 2008; Gao et al., 2015; Zhang et al., 2016), functional near-infrared spectroscopy (fNIRS; Plichta et al., 2011; Kovelman et al., 2012; Dewey and Hartley, 2015), and multimodal imaging (i.e., concurrent fNIRS, fMRI, and/or MEG; Kovelman et al., 2015; Corsi et al., 2019) to identify this process. In these studies, the complexities of brain responses evoked by the perception of sounds have been investigated to improve the quality of life.

Griffiths and Warren (2004) argued that analyzing auditory objects in the two-dimensional space (frequency and time) rather than one-dimensional space (frequency or time) is more meaningful, and thus acoustic experiences that produce two-dimensional images need to be investigated. But, they did not provide specific sound categories in their work. Theunissen and Elie (2014) showed that natural sounds facilitate the characterizations of the stimulus-response functions for neurons than white noise or simple synthetic sounds. Salvari et al. (2019) demonstrated significant activation and interconnection differences between natural sounds and human-made object sounds (music and artificial sounds) in the prefrontal areas using MEG. However, there were no significant differences between music and artificial sounds. Liu et al. (2019) demonstrated that predictions of tuning properties of putative feature-selective neurons match data from the marmoset’s primary auditory cortex. Also, they showed that the exact algorithm of marmoset’s call classification could successfully be applied in call classification in other species.

Identifying the sound that a person hears using a brain-computer interface (BCI) enables us to know what the person is hearing. The more diverse sounds a BCI device can discern, the more variant conditions are identified. For those who have lost vision, sound may be an alternative tool to communicate with other people in a non-contact way. If it is possible to classify more sounds, we can increase the control commands for an external device. Zhang et al. (2015) have researched decoding brain activation from multiple sound categories in the human temporal cortex. Seven different sound categories (English, non-English, vocal, Animal, mechanical, music, and nature) were used for classification in their fMRI work. They reported sound-category-selective brain maps showing distributed patterns of brain activity in the superior temporal gyrus and the middle temporal gyrus. However, analyses of such responses were hampered by the machine noise produced during fMRI experiments (Scarff et al., 2004; Fuchino et al., 2006).

fNIRS is a non-invasive brain imaging method that uses near-infrared light (700–900 nm) to penetrate the head and records oxygenation changes in the cerebral blood flow. fNIRS is a promising method for analyzing sound and speech processing. Compared to fMRI, fNIRS measurement is not noisy, and such measurements can be made in an environment more conducive to infant studies. Owing to these advantages, fNIRS shows significant potential for real-time brain monitoring while the subject is moving. According to fNIRS analyses, newborns consistently exhibit a strong hemodynamic response to universally preferred syllables, which suggests that the early acquisition and perception of language can be detected using categorical linguistic sounds (Gomez et al., 2014). The applications of this technology have the potential to provide feedback for speech therapy or in the tuning of hearing aid devices (e.g., cochlear implants) at an early stage of development based on brain recordings (Mushtaq et al., 2020). Several groups have demonstrated fNIRS use for measuring brain responses in deaf children with cochlear implants (Sevy et al., 2010; Pollonini et al., 2014).

In fNIRS applications, classification has been used in lie detection (Bhutta et al., 2015), drowsiness detection (Khan and Hong, 2015), mental workload detection (Herff et al., 2014), brain disease (Yoo et al., 2020), and the fNIRS-EEG-based hybrid BCI (Yuan et al., 2019; Lin et al., 2020). In fMRI applications, classification has also been used to decode the brain responses evoked by sight (Kohler et al., 2013; Smith, 2013) and sound (Staeren et al., 2009; Zhang et al., 2015). Lotte et al. (2007) and Pereira et al. (2009) reviewed the classification algorithms for EEG and fMRI data, respectively.

Recently, numerous studies have focused on improving classification accuracy by applying deep learning technology to brain signal classification, in addition to artificial neural networks (ANNs; Badai et al., 2020; Flynn et al., 2020). Convolutional neural networks (CNNs) and recurrent neural networks (RNNs) are representative forms of ANNs. The CNN is robust in processing large image data sets (Lee et al., 2020). It has been widely implemented in brain signal processing, including fMRI (Erturk et al., 2020), deep brain stimulation (Kakusa et al., 2020), and EEG (Lun et al., 2020). Besides, CNNs have been used to diagnose brain diseases in the fNIRS domain (Xu et al., 2019a; Yang et al., 2019, 2020). RNNs are capable of predicting and classifying sequential data. They have been widely applied in robotics for various purposes and systems such as obstacle avoidance control (Xu et al., 2019b; Zheng et al., 2019; Zhao et al., 2020), self-organizing robot control (Smith et al., 2020), collision-free compliance control (Zhou et al., 2019), dynamic neural robots (Tekulve et al., 2019), and self-driving system (Chen et al., 2019). Recently, RNNs have achieved impressive results in detecting seizures (Sirpal et al., 2019), brain injuries (Ieong et al., 2019), and pain (Hu et al., 2019b), as well as in discriminating attention-deficit hyperactivity disorder (Dubreuil-Vall et al., 2020).

Long short-term memory (LSTM) is a type of RNN incorporating a progressive model (Hochreiter and Schmidhuber, 1997). Compared to RNN, LSTM networks possess a “gate” to reduce the vanishing gradient problem and allow the algorithm to more precisely control the information that needs to be retained in memory and the information that must be removed. LSTM is also considered superior to RNNs when handling large sequences of data. Additionally, compared to CNN, it exhibits better performance in classifying highly dynamic nonlinear time-series data such as EEG data (Tsiouris et al., 2018; Li et al., 2020).

This study aims to develop a communication method for the completely paralyzed with no vision. We identify the sound that a person hears by measuring task-evoked hemodynamic responses from the auditory cortex: In our early work (Hong and Santosa, 2016), four sound categories were classified. When remotely communicating with people without vision, visual or motor cortex-based BCIs may not be applicable. Sound will be a vital tool to communicate. In this article, we increased the number of sound categories from four to six. The more diverse sounds are classified, the more variant conditions are identified. Eventually, we can diversify the control commands to operate an external device. Sound-based BCI using audio stimuli is promising because we can use such audio signals in our daily lives (i.e., a passive BCI is possible). In this article, auditory-evoked HRs are measured using fNIRS, and subsequently, LSTM is applied to analyze fNIRS’ ability to distinguish individual sounds out of six classes.

## Materials and Methods

### Subjects

A total of 18 subjects participated in the experiment (age: 26.89 ± 3.49 years; seven females, two left-handed). All subjects had normal hearing and no history of any neurological disorder. All subjects were informed about the nature and purpose of the respective experiments before obtaining their written consent. For the experiment, each subject lay down on a bed. All subjects were asked to remain relaxed, close their eyes, and avoid significant body movements during the experiment. The subjects were asked to listen attentively to various audio stimuli and guess the category of each stimulus. After the experiment, all participants were asked to explain verbally whether they could precisely distinguish what they heard. The fNIRS experimentation was done on healthy subjects and the entire experimental procedure was carried out in accordance with the Declaration of Helsinki and guidelines approved by the Ethics Committee of the Institutional Review Board of Pusan National University.

### Audio Stimuli

The audio stimuli consisted of six different sound categories selected from a popular website (http://www.youtube.com). As shown in Table 1, the first and second categories entailed speech in English and other languages (non-English). The subjects were Indonesian, Korean, Chinese, Vietnamese, and Pakistani. Each participant had a common recognition of English but failed to recognize the other languages. The third and fourth categories were annoying sounds and nature sounds. The fifth category was a segment of classical music (Canon in D by Pachelbel). The sixth category was gunshot sounds at a frequency of 1 Hz. Each category consisted of six different sounds (except the gunshots, which had the same repeated sound). Each subject was exposed to 36 trials (i.e., six sound categories × six trials). The audio stimuli were presented in a pseudo-randomized order. Each stimulus consisted of 10 s of the sound followed by 20 s of silence.

**Table 1:** Audio categories (M: male, F: female).

Trial	Human vocal hearing		Nonvocal hearing			
	English	Non-English	Annoying sound	Nature sound	Music	Gunshot
1	M	Russian (F)	Baby cry	River	Canon in D	10 times
2	F	German (F)	Car alarm	Forest (day time)	Canon in D	10 times
3	M	French (F)	Police siren	Rain	Canon in D	10 times
4	MF*	Bulgarian (MF*)	Horror sound	Jungle	Canon in D	10 times
5	F	Italian (MF)	Male scream	Ocean waves	Canon in D	10 times
6	F	Japanese (F)	Nuclear alarm siren	Waterfall	Canon in D	10 times

*MF denotes male–female conversation.

Additionally, pre- and post-trials of classical music were added (to avoid sudden hearing), neither of which was included in the data processing. Accordingly, the entire fNIRS recording lasted for approximately 19 min. All audio stimuli were digitally mixed using the Adobe Audition software (MP3-format file: 16-bit quantification, 44.1 kHz sampling, stereo channel) and normalized to the same intensity level. Active noise-cancellation earbuds (Sony MDR-NC100D) were utilized for acoustic stimulation of all subjects with the same sound-level setting. After each fNIRS recording session, all subjects reported that they could accurately distinguish the sound among the sound categories for all trials.

### fNIRS Measurements

**Figure 1:**
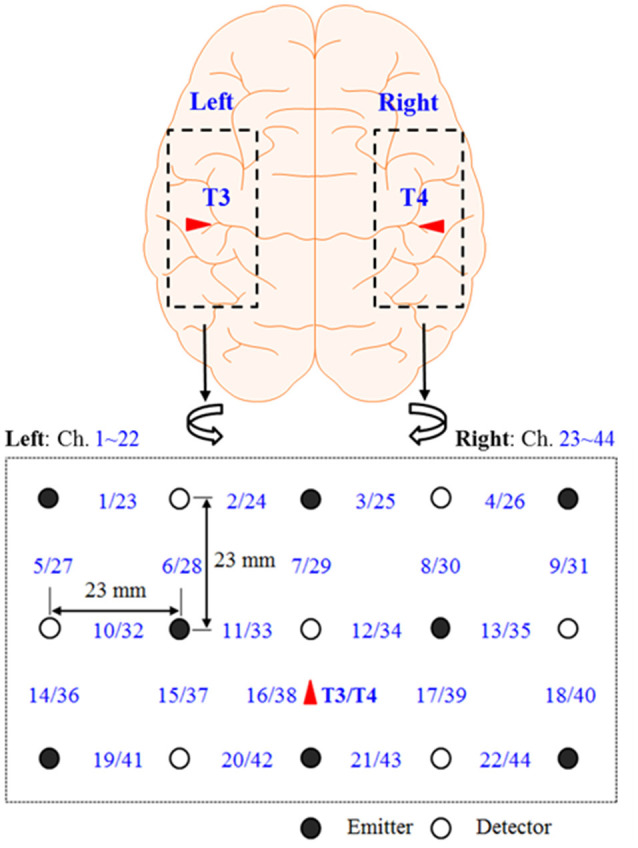
Optode configuration: the numbers represent the measurement channels, where Chs. 16 and 38 coincide with T3 and T4 locations in the International 10–20 system (Hong and Santosa, 2016).

Figure 1 shows the continuous-wave fNIRS system’s optode configuration (DYNOT: DYnamic Near-infrared Optical Tomography; NIRx Medical Technologies, Brooklyn, NY, USA) for bilateral imaging of the auditory cortex in both hemispheres. The emitter–detector distance was 23 mm, while the sampling rate was set to 1.81 Hz at two wavelengths (760 and 830 nm). The optode configuration consisted of 3 × 5 arrays (eight emitters and seven detectors) with 22 channels for each hemisphere. The two 22-channel sets were placed on the scalp, covering the left (Chs. 1–22) and right (Chs. 23–44) temporal lobes. According to the International 10-20 System, Chs. 16 and 38 were placed at T2 and T4, respectively (Santosa et al., 2014). In the left hemisphere, both Broca’s area and Wernicke’s area were covered by this configuration. Finally, the lights in the room were switched off to minimize signal contamination from ambient light sources during the experiments.

### Preprocessing

The optical data of two wavelengths were converted into relative oxy-hemoglobin (HbO) and deoxy-hemoglobin (HbR) concentration changes using the modified Beer-Lambert law (Hiraoka et al., 1993) using MATLAB^TM^ (2020b, MathWorks, USA). Owing to the uniform emitter-detector distance, constant values of differential path-length factors were used for all channels (i.e., *d* = 7.15 for *λ* = 760 nm and *d* = 5.98 for *λ* = 830 nm). In previous studies, HbO data were activated significantly higher than HbR for given stimuli. Therefore, only HbO data were processed in this study. The HbO data were filtered to remove physiological and artificial noises using the fifth-order Butterworth band-pass filter with cutoff frequencies of 0.01 Hz and 0.1 Hz. The filtered data was chopped for each trial.

### Feature Extraction for Support Vector Machine

The mean, slope, kurtosis, and skewness values of HbO signals were used as support vector machine (SVM) features. SVM classification was performed twice: One for “within-subject” and the other for “across-subject.” Within-subject classification is a standard classification method for the fNIRS study. Considering the total number of trials for one subject was 36, 6-fold cross-validation was performed for each subject. For the across-subject classification, we used the entire data set. In this case, the total number of trials was 648 (i.e., multiplication of the number of subjects and the number of trials. Ten-fold and leave-one-out cross-validation were performed for the across-subject classification. Training and testing sets were divided randomly by MATLAB^TM^ function *cvpartition* for cross-validation. The same data partitions were used for SVM and LSTM.

### LSTM

A recurrent neural network (RNN) is a type of artificial neural network wherein hidden nodes are connected with directional edges as a directed cycle. It is well known as an effective tool to process sequential data such as voice and handwriting. The RNN has the following structure (Hochreiter and Schmidhuber, 1997):

(1) $$y_{t}=W_{hy}h_{t}+b_{y},$$

(2) $$h_{t}=\tanh\;(W_{hh}\;h_{t-1}+W_{xh}x_{t}+b_{h}),$$

where *y_t_* indicates the output of the present state; subscript *t* is the discrete time step; *W_hy_*, *W_hh_*, and *W_xh_* are the parameters from layer to layer; *b_y_* is the bias of the output *y*; *h_t_* is the hidden state vector; *x_t_* is the input vector; and *b_h_* is the bias of the hidden state vector *h*.

The LSTM is a special kind of recurrent neural network, compensating for the vanishing gradient problem. It has a structure of cell-states in the hidden state of RNN. The basic formulas for LSTM are as follows.

(3) $$f_{t}=\sigma (W_{xf}x_{t}+W_{hf}h_{t-1}+b_{f}),$$

(4) $$i_{t}=\sigma (W_{xi}x_{t}+W_{hi}h_{t-1}+b_{ο}),$$

(5) $${ο}_{t}=\sigma (W_{xο}x_{t}+W_{hο}h_{t-1}+b_{ο}),$$

(6) $$g_{t}=\tanh(W_{xg}x_{t}+W_{hg}h_{t-1}+b_{g}),$$

(7) $$c_{t}=f_{t}\circ c_{t-1}+i_{t}\circ g_{t},$$

(8) $$h_{t}={ο}_{t}\circ \tanh(c_{t}),$$

where *f_t_* is the activation vector of forgetting gate to forget past information; *i_t_* is the activation vector of input gate to memorize the current information; *o_t_* is the activation vector of output gate; *g_t_* is the activation vector of the cell input; *c_t_* is the cell state vector; *W_xf_*, *W_hf_*, *W_xi_*, *W_hi_*, *W_xo_*, *W_ho_*, *W_xg_*, and *W_hg_* are the weight matrices of the input and recurrent connections; *b_f_*, *b_i_*, *b_o_*, and *b_g_* are the parametric bias vectors; and ° is the Hadamard product. In the LSTM networks, cell state and hidden state are calculated recursively.

**Figure 2:**
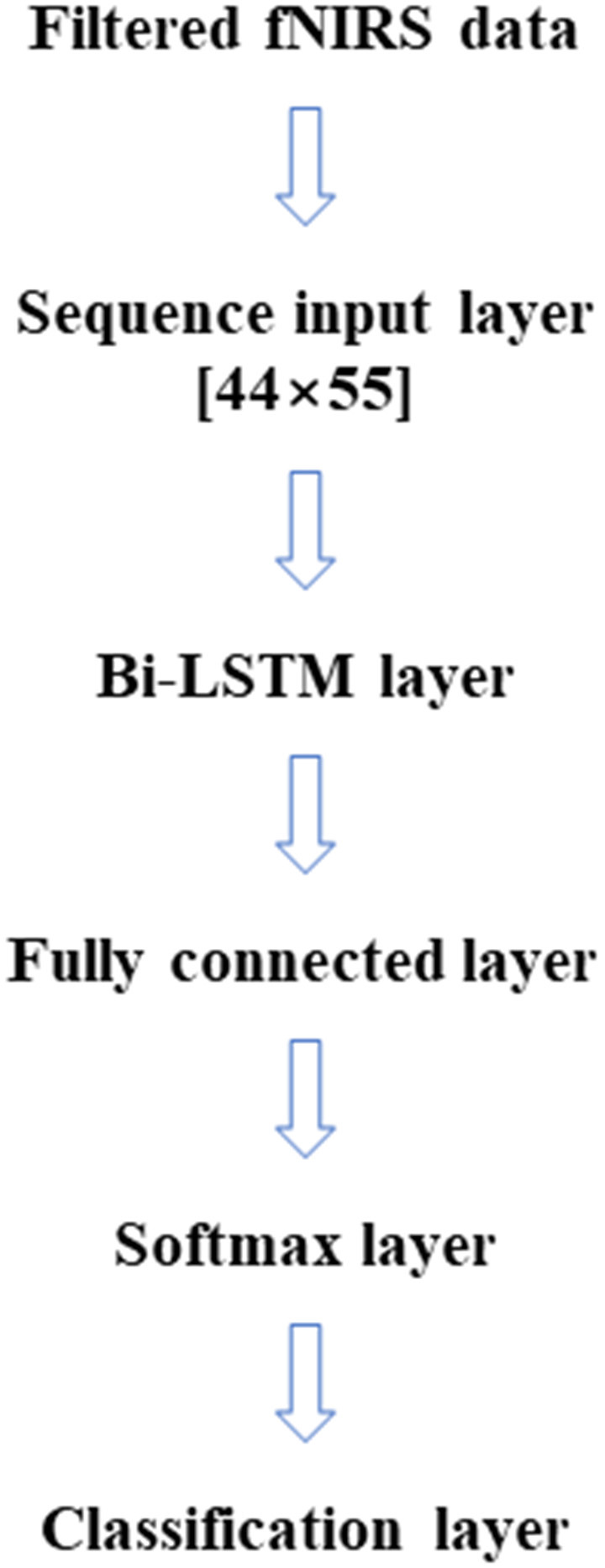
Simple bi-LSTM model for classification.

In this article, LSTM networks are applied in two ways, like the two cases (within-subject, across-subject) in the SVM classification. Figure 2 represents the LSTM networks used in this article. First, for within-subject classification, a bi-LSTM layer of eight hidden layers was used with two maximum epochs and three mini-batch sizes (Kang et al., 2020). Second, the bi-LSTM layer of 16 hidden layers was used for across-subject classification with three maximum epochs and three mini-batch sizes, see Table 2. The number of hidden layers, maximum epoch, and mini-batch size were selected to avoid overfitting (Sualeh and Kim, 2019). Additionally, a bi-LSTM layer of 256 hidden layers was examined for across-subject classification (to compare with 16 hidden layers). Also, 6- and 10-fold and leave-one-out cross-validations were performed in the same way as SVM.

**Table 2:** A single bi-LSTM network.

bi-LSTM structure			
	Across-subject		Within-subject
	10-fold	Leave-one-out	
Input size	44 × 55		44 × 55
Training data set	584	612	30
Testing data set	64	36	6
The number of hidden layers	16		8

## Results

**Figure 3:**
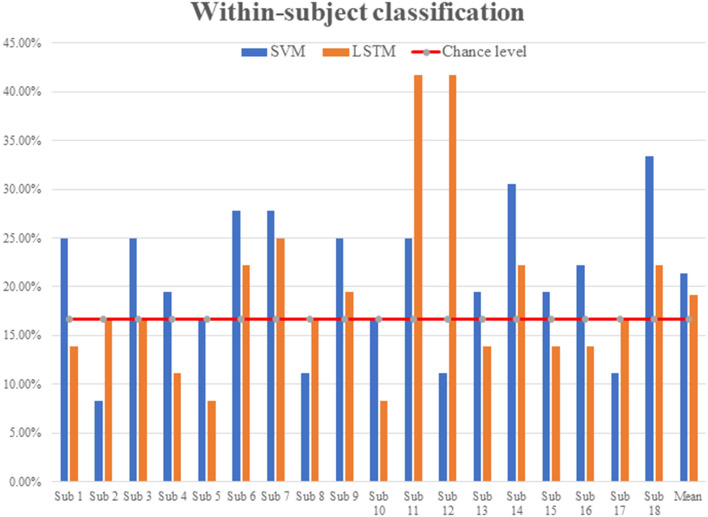
Within-subject classification accuracies (SVM vs. LSTM).

In the experiment, a total of 18 subjects listened to six repetitions of each of the six categories of sound stimuli (36 total trials). The six categories were English (E), non-English (nE), nature sounds (NS), music (M), annoying sounds (AS), and gunshot (GS). The within-subject classification accuracies were 21.35 ± 6.71% for SVM and 19.14 ± 9.16% for LSTM, respectively; see Figure 3.

**Figure 4:**
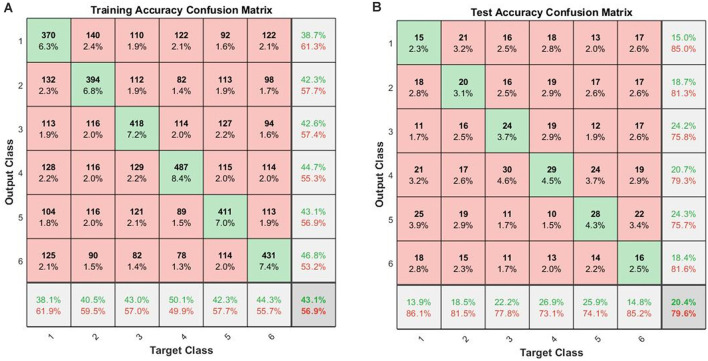
Across-subject classification accuracies (LSTM): **(A)** confusion matrix for training, **(B)** confusion matrix for testing (Class 1: English; Class 2: non-English; Class 3: Nature sound; Class 4: Music; Class 5: Annoying sound; Class 6: Gunshot).

When the cross-validations of SVM and LSTM were performed separately, the accuracies of the 10-fold across-subject classification with 16 hidden layers were 16.83 ± 3.90% for SVM and 20.38 ± 4.63% for LSTM, respectively. Figure 4 shows the confusion matrices for training and testing for the 10-fold across-subject classification. The hypergeometric *p*-values were calculated using the confusion matrix in Figure 4B. The *p*-values were 0.3745 (E), 0.3123 (nE), 0.0232 (NS), 0.0946 (M), 0.0129 (AS), and 0.3701 (GS). For a fair comparison between SVM and LSTM, we repeated the 10-fold cross-validation using the same data partitioning using the same code. In this case, the results were 15.73 ± 3.00% for SVM and 21.44 ± 4.57% for LSTM. Henceforth, we could not find a significant difference between the two cases regarding the data partitioning method.

**Figure 5:**
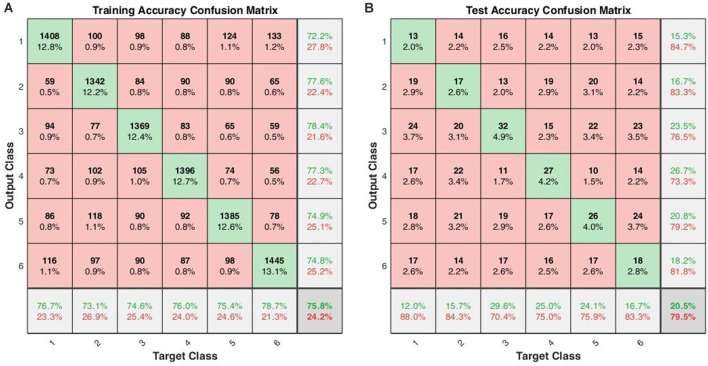
Across-subject classification accuracies (leave-one-out validation of the LSTM with 16 hidden layers): **(A)** confusion matrix for training, **(B)** confusion matrix for testing (Class 1: English; Class 2: non-English; Class 3: Nature sound; Class 4: Music; Class 5: Annoying sound; Class 6: Gunshot).

Using the same data partitioning for SVM and LSTM, we also performed the leave-one-out across-subject classification. The results were 16.83 ± 3.90% for SVM and 20.52 ± 6.15% for LSTM. Figure 5 shows the confusion matrices for training and testing in the leave-one-out case. The hypergeometric *p*-values were calculated using the confusion matrix in Figure 5B. The *p*-values were 0.4274 (E), 0.5488 (nE), 0.0129 (NS), 0.0036 (M), 0.1078 (AS), and 0.3769 (GS). The low *p*-values indicate that the classifier could successfully classify the sounds.

LSTM showed better accuracies in the across-subject classification but worse accuracies in the within-subject classification: This result was somewhat unexpected in comparison to the four-sound case (Hong and Santosa, 2016). It suggests that, in the six categories case, the subjects heard too many sound-categories, and they had difficulty in distinguishing them. Overall, when there are many hidden layers in the classifier, training becomes better than when there are few, but overfitting to the training data occurs. It is noted that the across-subject classification accuracies of LSTM with 256 hidden layers were 99.9% for training and 23.15% for testing, which is an overfitting case.

## Discussions

The previous studies in the literature have shown that various sound categories were processed differently in the brain. Staeren et al. (2009) showed that different sound categories evoked significant BOLD responses in a large expanse of the auditory cortex, including bilaterally the Heschl’s gyrus, the superior temporal gyrus, and the upper bank of the superior temporal sulcus. Zhang et al. (2015) revealed that sound category-selective brain maps demonstrated distributed brain activity patterns in the superior temporal gyrus and the middle temporal gyrus. Plichta et al. (2011) reported that pleasant and unpleasant sounds increased auditory cortex activation than neutral sounds in their fNIRS research. Our results showed that nature sounds, music, and annoying sounds were classified better than other categories. Nature sounds and music are considered pleasant sounds, and annoying sounds are unpleasant sounds (Plichta et al., 2011). Classifying emotionally-neutral sounds (i.e., E, nE, and GS) “individually” from other categories is considered difficult.

Deep learning algorithms have been developed to increase classification accuracy and stability (Shan et al., 2019; Park and Jung, 2020; Sung et al., 2020). RNNs have been developed to improve their performance likewise; memristor-based RNNs (Yang et al., 2021), chaotic delayed RNNs with unknown parameters and stochastic noise (Yan et al., 2019), reformed recurrent Hermite polynomial neural network (Lin and Ting, 2019). The developed RNNs have been applied in various brain imaging techniques (Hu et al., 2019a; Plakias and Boutalis, 2019). Wang et al. (2019) achieved 98.50% of classification accuracy by convolutional RNNs for individual recognition based on resting-state fMRI data. Qiao et al. (2019) proposed the application of bi-LSTM to decoding visual stimuli based on fMRI images from the visual cortex. The classification accuracies were 60.83 ± 1.17% and 42.50 ± 0.74% for each subject in five categories. The number of training samples and validation samples were 1,750 and 120 for each subject. Compared with the existing research, a limitation of this research is the small size of the data set.

The conventional classification technique is the process of distinguishing data from a set of categories based on a training data set on the category classes of which are known (Klein and Kranczioch, 2019; Pinti et al., 2019). The individual observations are analyzed into a set of features selected and executed by a classifier (Khan et al., 2014). In a more detailed process, a classifier is a function that takes the values of various features. For example, the mean, slope, skewness, and kurtosis values of HbO and HbR signals from individual trials can be used as the feature set (Tai and Chau, 2009). In our previous research (Hong and Santosa, 2016), decoding four-class sounds categories using fNIRS showed the 46.17 ± 6.25% (left) and 40.28 ± 6.00% (right) accuracies using LDA, while showing 38.35 ± 5.39% (left) and 36.99 ± 4.23% (right) using SVM. In the previous study, the classification was performed with the following steps: filtering, selecting region of interest, feature extraction from the region of interest, and classification. In the current study, to compare with the proposed method, the conventional classification technique was applied with the following steps: filtering, feature extraction from all channels, and classification. For LSTM networks, only filtering has been applied before classification.

The LSTM network may indirectly extract unstructured features from the data, and the network’s weighting factors are optimized during the training session. The network can be trained only after simple filtering. The results showed that SVM is better for within-subject classification and LSTM is better for across-subject classification. It seems that fNIRS data involve different physiological data per subject, but this physiological difference is not removed with simple filtering. Also, the sample sizes of within-subject classification were 30 for training and six for validation. The sample sizes of across-subject classification were 584 for training and 64 for validation. Additionally, there are no significant differences between 10-fold and leave-one-out validations. The dataset of 10-fold validation might use the same subject’s data in either training or testing. The size of the training data set was bigger in the leave-one-out validation. According to this result, if the sample size increases, the LSTM network would show better performance than the conventional method (SVM). Also, if we have enough training data, it would be enough for ignoring the subjects’ physiological differences in the LSTM network. The LSTM network with 256 hidden layers showed slightly better performance than others, but the network overfitted with the training data set rapidly. Simplifying the data classification time can contribute to the commercialization of future diagnostics using fNIRS or BCI technology, given that it can reduce the classification time. Although the results in this study are not outstanding, it is worthwhile to show the potential of deep learning-based fNIRS signal classification technology.

## Conclusion

This article aimed to identify hearing sounds using the HRs from the auditory cortices. The proposed audio-signal-based BCI is to be used for completely paralyzed people, for whom visual or motor cortex-based BCI may not be suitable. In this study, we used fNIRS signals evoked by audio-stimuli from multiple sound-categories. Compared with the conventional method, the LSTM-based approach could decode the brain activities without heavy pre-processing of the data, such as regression, feature selection, and feature extraction. Though the LSTM network’s performance was a little higher than the chance level, it is noteworthy that we could classify the six sounds virtually without defining the region of interest and feature extraction. The approach using audio stimuli is promising for a passive-type BCI using ordinary sounds in our daily lives. This study has a limitation on the number of data, which needs to be improved in the future.

## Data Availability Statement

The datasets generated in this article are available on request to the corresponding author.

## Ethics Statement

The studies involving human participants were reviewed and approved by Ethics Committee of the Institutional Review Board of Pusan National University. The patients/participants provided their written informed consent to participate in this study.

## Author Contributions

S-HY conducted the literature review and wrote the first draft of the manuscript. HS obtained the experimental data and initiated the work. C-SK participated in the revision of the manuscript. K-SH conceived the idea, corrected the manuscript, and finalized the work. All the authors have approved the final manuscript.

## Conflict of Interest

The authors declare that they have no conflict of interest. This research was conducted in the absence of any commercial or financial relationship that could be construed as a potential conflict of interest.
